# When “AA” is long but “A” is not short: speakers who distinguish short and long vowels in production do not necessarily encode a short–long contrast in their phonological lexicon

**DOI:** 10.3389/fpsyg.2015.00438

**Published:** 2015-04-24

**Authors:** Kateřina Chládková, Paola Escudero, Silvia C. Lipski

**Affiliations:** ^1^Amsterdam Center for Language and Communication, University of AmsterdamAmsterdam, Netherlands; ^2^The MARCS Institute, University of Western SydneySydney, NSW, Australia; ^3^Jean-Uhrmacher-Institute for Clinical ENT Research, University of CologneCologne, Germany

**Keywords:** phonological representations, vowel length, mismatch negativity, duration processing, short–long contrast

## Abstract

In some languages (such as Dutch), speakers produce duration differences between vowels, but it is unclear whether they also encode short versus long speech sounds into different phonological categories. To examine whether they have abstract representations for ‘short’ versus ‘long’ contrasts, we assessed Dutch listeners’ perceptual sensitivity to duration in two vowel qualities: [a] and [ɑ], as in the words *maan* ‘moon’ and *man* ‘man,’ which are realized with long and short duration respectively. If Dutch represents this phonetic durational difference as a ‘short’–‘long’ contrast in its phonology, duration changes in [a] and [ɑ] should elicit similar neural responses [specifically, the mismatch negativity (MMN)]. However, we found that duration changes evoked larger MMN amplitude for [a] than for [ɑ]. This finding indicates that duration is phonemically relevant for the *maan*-vowel that is represented as ‘long,’ while it is not phonemically specified for the *man*-vowel. We argue that speakers who in speech production distinguish a given vowel pair on the basis of duration may not necessarily encode this durational distinction as a binary ‘short’–‘long’ contrast in their phonological lexicon.

## Introduction

Phonological representations (such as phonemes or phonological features) are stored functional entities of speech sounds. They can be described as abstract correspondents of speech sounds that function at a discrete linguistic level free from the actual physical (i.e., auditory or articulatory) properties of speech signals. For instance, the physical dimension of the first formant (F1) is universally used to contrast vowels (Maddieson, 1984). In line with that, language users encode the relevant F1 differences in their grammar in terms of discrete vowel height categories, as reflected in listeners’ pre-attentive perceptual processing (e.g., Scharinger et al., 2011; pre-attentive processing is automatic processing of speech sounds without listeners’ explicit attention to the sounds and without any overt response required from them).

With respect to linguistic representations for *vowel length*, there are two types of languages. On the one hand, there are *quantity languages* (such as Czech, Estonian, Finnish, or Japanese) that encode vowel duration into abstract linguistic categories, which means that phonologically short vowels are produced, i.e., phonetically realized, with short duration, while long vowels are phonetically realized with long duration. In quantity languages, a short and a long member of a vowel contrast are primarily distinguished by duration and secondarily by spectral properties (the long member of the pair being slightly more peripheral than the short one). On the other hand, there are *non-quantity languages* (such as Greek, Portuguese, or Spanish) that do not encode vowel duration into abstract linguistic categories, which means that phonetically short and long vowels are not realizations of different phonological categories. To date, however, for some languages it is not clear whether they have abstract phonological categories for vowel length, that is, whether speakers of these languages represent physical duration differences between vowels in terms of a phonological ‘short’–‘long’ contrast. One of such unresolved cases is Dutch.

The phonological status of vowel length in Dutch has been debated for decades and remains a question (for a recent review, see Botma and van Oostendorp, 2012). One of the reasons why the relation between phonetic duration and mental phonological representations for length is not clear in Dutch is because phonological analyses, speech production and speech perception studies provide conflicting evidence.

Northern Standard Dutch has 15 vowels, all produced with different spectral properties: nine monophthongs /i I y Y ε aː ɑ ɔ u/, three diphthongs /εI œy ɔ u/, and three ‘potential’ diphthongs /eː øː oː/ (realized as [ei øy ou], respectively). Formal analyses of the Dutch vowel system that consider vowel length to be part of Dutch phonology (e.g., Moulton, 1962; Zonneveld, 1993) describe /I Y ε ɑ ɔ/ as short vowel phonemes, and /i y u aː eː øː oː εI œy ɔ u/ as long vowel phonemes. One reason for such phonological description is, for example, the fact that the two different groups of vowels occupy different syllabic positions: phonologically long vowels can occur in open syllables (i.e., syllables ending in a vowel), while phonologically short vowels cannot. For instance, the vowel /i/ is considered a long counterpart of the phonologically short vowel /I/, as is /y/ to /Y/, as is /eː/ to /ε/, or /aː/ to /ɑ/. In line with their phonological length membership, in Dutch, consonant (C)+/i/ or C+/aː/ is allowed as a syllable, while C+/I/ or C+/ɑ/ is not: the latter two have to be followed by another C.

Phonetically, the six diphthongs and /aː/ are usually produced with a long duration, while the remaining eight monophthongs /i I y Y ε ɑ ɔ u/ are produced with a short duration (Adank et al., 2004). A discrepancy between phonological analyses and phonetic reality arises: the vowels described as phonologically “long” are not all produced with long duration (which is what one finds in quantity languages that unequivocally employ a short–long contrast in their phonology). Specifically, even though /i/, /y/, and /u/ are phonetically short, phonological theories describe these vowels as long. Thus, Dutch speakers do not seem to use duration consistently across all phonologically short–long contrasts. Some of the phonological short–long contrasts, such as /I/-/i/, are in speech production distinguished solely by their spectral properties and not by duration. Moreover, even those short–long contrasts that are produced with different durations at the phonetic level, such as /aː/-/ɑ/ or /eː/-/ε/, still entail a considerable spectral difference. It is therefore not clear whether Dutch speakers encode durational differences into abstract short versus long categories.

A review of speech *perception* research can provide a deeper insight into whether Dutch speakers have phonological representations for vowel duration differences and what these representations are. The contrast that has been given wide attention in previous speech perception research is the /aː/-/ɑ/ contrast (as in the words *maan* ‘moon’ and *man* ‘man’), perhaps because of the large durational difference between the two vowels: /aː/ is usually produced with a duration twice as long as that of /ɑ/ (Nooteboom and Doodeman, 1980; Adank et al., 2004). Our reasoning is that if Dutch phonology encodes duration in terms of abstract categories ‘short’ and ‘long,’ Dutch listeners’ perception of the duration difference between /aː/ and /ɑ/ should reflect such phonological encoding of length. In that respect, in an overt behavioral vowel classification task, Escudero et al. (2009) found that Dutch listeners almost neglected the duration differences between /aː/ and /ɑ/ and instead relied on spectral properties to distinguish these two vowels (for a similar finding see also van Heuven et al., 1986). Auditorily, however, Dutch listeners clearly do detect durational changes among tokens of /aː/, as shown by Lipski et al. (2012) where duration changes between [aː] and [a] evoked a similar pre-attentive auditory response as did spectral changes between [a] and [ɑ]. A difference between Escudero et al. (2009) and Lipski et al.’s (2012) stimulus sets should be noted here: the former tested durational reliance for both /aː/ and /ɑ/, while the latter did so for only /aː/.

Chládková et al. (2013) also tested the perceptual processing of duration in the vowel /aː/ and, similarly to Lipski et al. (2012), found that Dutch listeners are indeed sensitive to duration of /aː/. Moreover, Chládková et al. (2013) compared Dutch listeners’ processing of duration in [a] to their processing of duration in a non-native vowel [ɣ]. In order to test whether Dutch has phonological representations for length or not, that is, whether Dutch is a quantity language or a non-quantity language, Dutch listeners’ neural responses to duration were compared to those of listeners from both types of languages (Czech and Spanish, respectively). The neural responses of the Dutch participants differed from the other language types depending on the spectral properties of the vowel they heard. For [a], Dutch listeners exhibited large sensitivity to duration changes comparable to that of Czech and larger than that of Spanish listeners. In contrast, for [ɣ], Dutch listeners had a smaller sensitivity to duration differences than both Czech and Spanish listeners. Thus, on the one hand, Dutch listeners differed from Czech listeners (who encode length in their phonology) in their perception of duration in [ɣ], which indicated that Dutch listeners might not have abstract representations for vowel length in vowel qualities other than [a]. On the other hand, no significant difference for duration changes was detected between [a] and [ɣ] *within* Dutch listeners, indicating that the Dutch sensitivity to duration might not differ across different vowel qualities. Given the latter null result, no reliable conclusion could be drawn regarding the phonological role of vowel length in Dutch. However, as the authors suggested, there was a vowel height confound that may have obscured any difference in duration processing between [a] and [ɣ], i.e., between a low and a mid vowel. Due to articulatory mechanisms, vowels differ intrinsically in duration: low vowels tend to be longer than mid vowels. It has been shown that this intrinsic vowel length difference between low and mid vowels affects the relative perception of duration changes (see Meister et al., 2011). In general, listeners may be universally more sensitive to a specific absolute duration change in an intrinsically short mid vowel than in an intrinsically long low vowel. If, however, a particular language (possibly Dutch) uses duration contrastively in low but not in mid vowels, this language-specific phonological effect may interfere with the above-mentioned psychoacoustic effect and obliterate a measurable difference in duration processing in mid versus low vowels.

In summary, the findings of studies on neural processing of duration differences show that Dutch listeners exhibit perceptual sensitivity to duration in the “long” vowel /aː/, while no pre-attentive data are available for duration processing in its “short” counterpart /ɑ/. Interestingly, a recent behavioral study on word recognition has shown that Dutch listeners identify a token of word-internal [a] (i.e., /aː/ with a short duration) as /ɑ/, but do *not* identify a token of word-internal [ɑː] (i.e., /ɑ/ with a long duration) as /aː/ (van der Feest and Swingley, 2011). Since Dutch listeners’ vowel identification in van der Feest and Swingley (2011) was more affected by duration changes in /aː/ than by duration changes in /ɑ/, one might argue that duration is perceptually relevant only for /aː/ and not for /ɑ/. However, listeners’ responses in behavioral tasks could be frequency-driven. Specifically, Dutch listeners might be able to discriminate [ɑ]-[ɑː] equally well as [a]-[aː], but identify only the former two as a single phoneme. That is, they may be less likely to overtly classify [a] and [aː] as a single phoneme, possibly because their experience tells them that [a] can occur as a realization of /ɑ/ in some Dutch dialects and consonantal contexts (see Benders, 2013, p. 91). In order to examine whether vowel duration is an equally strong perceptual cue across Dutch vowels, in the present study we carried out a direct comparison of Dutch listeners’ pre-attentive detection of duration changes for the two vowel categories /aː/ and /ɑ/. Using behavior independent measures allows us to collect data that are unaffected by listeners’ conscious decisions about stimulus category (driven by, e.g., their experience with various contexts or dialects) and will thus truly reflect these listeners’ perceptual sensitivity to duration changes in the stimuli.

The present study investigates whether Dutch listeners generalize their duration processing across the two low vowels [a] and [ɑ]. Note that these two vowels do not differ in height, and there is thus no confound of differential processing of duration in low versus non-low vowels that was present in Chládková et al. (2013). The aim is to test the hypothesis that Dutch listeners perceptually rely on duration to the same extent for both members of the vowel pair /aː/-/ɑ/, as should be the case if the binary length contrast ‘short’–‘long’ was represented in the phonology of Dutch.

The measure that we use to assess listeners’ perceptual sensitivity is the mismatch negativity (MMN). The MMN is a neural response elicited when infrequent deviations occur among frequently repeated sounds, and is modulated by linguistic experience: acoustic deviations that represent a phonemic change can elicit a stronger MMN response than those that do not represent a phonemic change (Näätänen et al., 1997, 2007; Sharma and Dorman, 2000; Phillips, 2001). Importantly for the purposes of the present study, the literature shows that the MMN elicited by duration changes is also affected by the listeners’ language background: duration changes yield a stronger MMN response in listeners from quantity languages than in those from non-quantity languages (e.g., Nenonen et al., 2003, 2005; Ylinen et al., 2006; Kirmse et al., 2008). For instance, the MMN elicited by the change in vowel duration between [ka] and [kaː] has larger amplitude in Finnish listeners, whose native language has length contrasts, than in Russian listeners, whose native language does not have phonological length contrasts (Nenonen et al., 2003).

**FIGURE 1:**
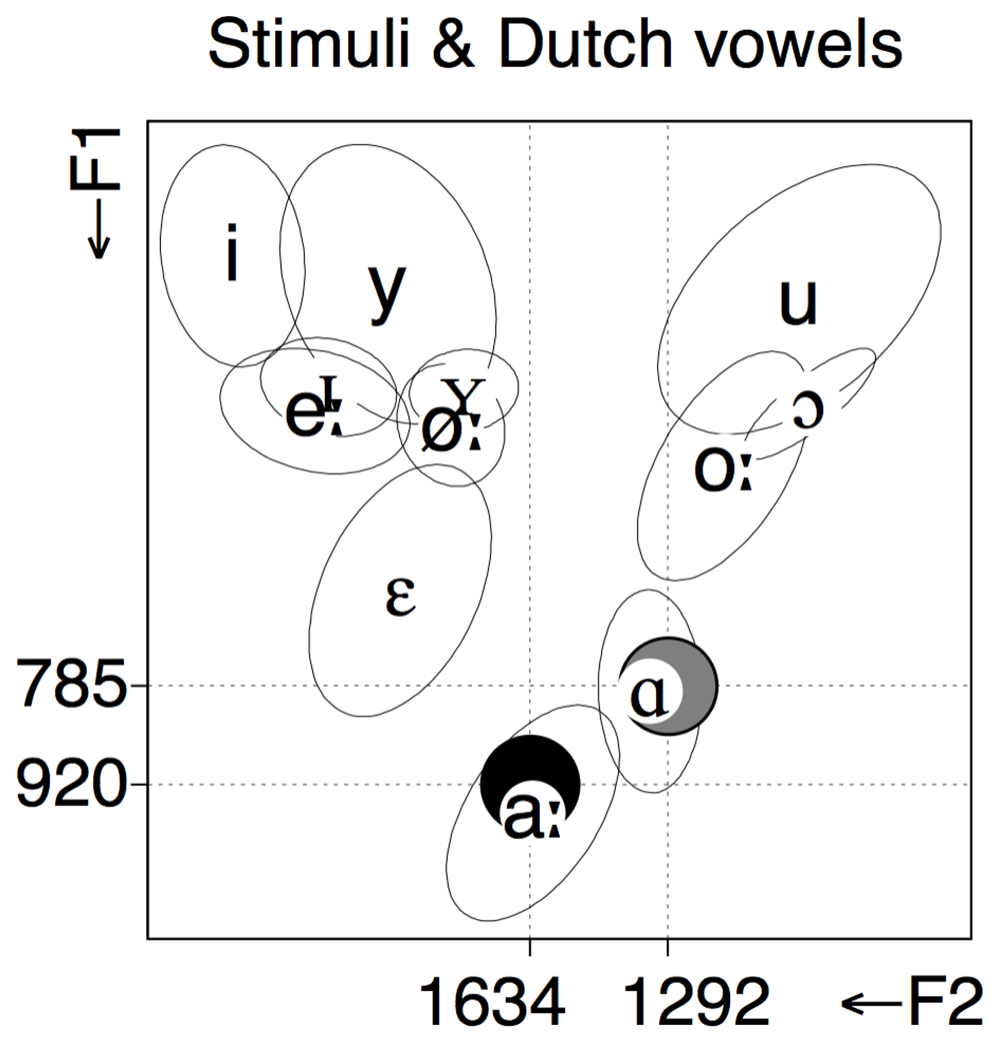
**F1 and F2 values of Randstad Dutch vowels produced by female speakers (van Leussen et al., 2011) and the two vowels produced by a female Estonian speaker that served as stimuli in the present study: [a] = black filled circle, [ɑ] = gray filled circle.** Phonetic symbols indicate the mean values of the Dutch vowels, ellipses show two standard deviations. Axes are scaled in Erb, marks are in Hz.

To test whether they encode their native /aː/-/ɑ/ contrast as a length contrast, we presented Dutch listeners with duration changes in isolated tokens of [a] and [ɑ], which resemble the quality of their native phonemes /aː/ and /ɑ/, respectively, as shown in **Figure 1**. If duration is phonemically relevant for /aː/ and not for /ɑ/, the change between [a] and [aː] should elicit a stronger MMN response than the change between [ɑ] and [ɑː]. If, on the other hand, duration is phonemically relevant for both /aː/ and /ɑ/, the change between [a] and [aː] and the change between [ɑ] and [ɑː] should elicit equally large MMN responses. Our predictions were tested with vowels produced in isolation (following previous studies such as Escudero et al., 2009; Lipski et al., 2012; Chládková et al., 2013; note that Ylinen et al., 2006 found similar language effects on MMN to duration changes across word-embedded and isolated vowels). Although vowel duration is relative to word and sentence context or speaking style, the present study used isolated vowels because the aim was to test the genuine perceptual encoding of vowel duration unaffected by top–down lexical or word frequency effects.

The present results will provide direct evidence to confirm or disconfirm the hypothesis that the Dutch language has a phonological vowel length contrast between a ‘short’ and a ‘long’ vowel category. That is, the present study will show whether or not speakers of a language that employs duration differences in the phonetic realization of a given vowel pair have equal perceptual sensitivity to vowel duration across both vowels in question, as should be the case if a binary short–long contrast were encoded as part of the linguistic representations in their mental lexicon.

## Materials and Methods

### Participants

Eighteen young healthy right-handed listeners took part. The MMN to duration changes was measured in two separate sessions: in one session participants listened to duration changes in [a], while in the other session they listened to duration changes in [ɑ]; the order of the two sessions was counterbalanced across subjects. Data from nine participants’ for [a] comes from the data reported in Chládková et al. (2013), i.e., 9 participants who were presented with [a] in that study’s first session (mean age at first session = 22.8, range = 19–26; three male). The nine participants listening to [ɑ] in their first session were newly recruited participants for the present study (mean age at first session = 22, range = 19–24; five male). For all participants, the second session was administered 10 months after the first session.

The participants were all monolingual Dutch native speakers from the Randstad area in the Netherlands. Seven additional participants were recruited for the first session: two of them had a large number of artifacts (i.e., more than 60% of artifact-contaminated deviant epochs) in the first session and were thus further excluded from the study, and five participants chose not to take part in the second session. Participants gave a written informed consent and were paid for participation. The study was approved by the ethical committee of the Faculty of Humanities, University of Amsterdam and conforms to the guidelines of the Declaration of Helsinki (2008).

### Stimuli

The stimuli were natural tokens of the Estonian vowels /æ/ and /ɑ/ produced in isolation (henceforth transcribed as [a] and [ɑ], respectively). The vowels were produced by a young native female speaker of Estonian. Subsequently, we selected one token of each vowel that had the most stable formant contour throughout its entire duration. The values of the first three formants were 920, 1634, and 2707 Hz for [a]; and 785, 1292, and 2675 Hz for [ɑ]. As shown in **Figure 1**, [a] is acoustically similar to Dutch /aː/, and [ɑ] is acoustically similar to Dutch /ɑ/. Estonian vowels were used because they are spectrally nearly identical to the respective two native Dutch vowels. At the same time, using Estonian offers the possibility of comparing duration processing in Dutch-like vowels to vowels that are unfamiliar to Dutch speakers and that were examined in a previous study (Chládková et al., 2013).

The selected [a] and [ɑ] tokens were subsequently manipulated with the time-domain pitch-synchronous overlap-and-add algorithm (Moulines and Charpentier, 1990) built in the program Praat (Boersma and Weenink, 2011) to yield 6 psychoacoustically equidistant duration steps: 118, 136, 157, 181, 208, and 239 ms. These six duration values replicated the duration values used in a previous ERP experiment with Dutch listeners (Chládková et al., 2013). The three short tokens (i.e., 118, 136, and 157 ms) overlap with the durations that have been commonly reported for (Northern) Dutch productions of the phoneme /ɑ/, while the three long tokens (i.e., 181, 208, and 239 ms) overlap with the durations of Dutch /aː/ (see Adank et al., 2007). That is, if Dutch speakers encode length in their phonology, and thus consider the /ɑ/-/aː/ contrast a length contrast, the three shortest durations of our stimulus set (i.e., of either the [a]- or the [ɑ]-series) would fall within their short category and the three longest durations would fall within their long category. The stimuli were presented in the categorical oddball paradigm (i.e., a many-to-many oddball paradigm which taps into abstract phonological representations, see Phillips, 2001), in which the three shortest items served as the stimuli representing the short category, while the three longest items served as the stimuli representing the long category.

### Procedure

As noted above, participants were tested in two sessions that took place on two different days: in one session, participants were presented with duration changes in [a], while in the other session they were presented with duration changes in [ɑ]. Each session consisted of two 30-min blocks of EEG-recording (block 1, block 2), with a 15-min break between blocks.

In one block, short vowels were the standard stimuli and long vowels were the deviants, while in the other block long vowels were standards and short vowels deviants. The order of blocks was counterbalanced across subjects. Each block started with 20 standards, followed by the oddball sequence, which contained 2000 standards and 300 deviants (100 deviants of each type); within an oddball sequence, the deviant category thus occurred with a probability of 15%. All three deviants and standards were evenly represented in both the deviant and the standard category. A deviant was always followed by 3–8 standards. The ISI was varied randomly in five equidistant steps between 800 and 932 ms. The stimuli were normalized for root-mean-square amplitude and presented at 60 dB SPL via a single loudspeaker placed in front of the participant at chin level, at a distance of 1 m.

Testing took place in a sound-attenuated laboratory at the University of Amsterdam. During stimulus presentation, participants watched a muted movie of their choice (originally spoken in Dutch) with subtitles in Dutch. Before the session started, participants were told they would hear Dutch vowels and were instructed to disregard them and just watch the movie.

### EEG Recording

The EEG signal was recorded from 64 active Ag-AgCl electrodes placed according to the International 10/20 placement in a cap (BioSemi) that was fitted to participant’s head size. We used seven external electrodes: placed on the nose, below and above the right eye, on the left and right temple, and on the right and left mastoid. The electrode offset was kept below ± 50 mV. The EEG was recorded at 8 kHz and subsequently downsampled to 512 Hz.

The EEG was oﬄine referenced to the nose channel. We removed slow drifts from the signal by subtracting from each channel a line so that the first and the last sample become zero. The data were band-pass filtered in the frequency domain with a low cut-off of 1 Hz (bandwidth = 0.5 Hz) and a high cut-off of 30 Hz (bandwidth = 15 Hz). The data were epoched from -100 to 700 ms relative to stimulus onset. For baseline correction the mean voltage in the 100-ms pre-stimulus interval was subtracted from each sample in the epoch. Artifact correction was done automatically (epochs in which the absolute amplitude exceeded ±75 μV at any channel were rejected) and by subsequent visual inspection. Participants with more than 60% of artifact-contaminated deviant epochs were excluded from further analysis; 120 was thus the minimum number of deviant events across which the mean deviant response was calculated for every participant.

### ERP Extraction and MMN Assessment

The MMN is a neural response that typically peaks between 100 to 250 ms after the onset of deviation, and has a negative deflection (Näätänen, 2001). It is measured in a difference waveform, which is derived by subtracting the average response to the standard from the average response to the deviant stimulus. The MMN reflects an automatic detection of deviation: if listeners detect a deviation, their ERP response to a stimulus when it occurs as a deviant differs from their ERP response to the same stimulus when it occurs as a standard (or on its own). In order assess the effects caused by listeners’ detecting the deviation rather than the effects caused by differential responses to two acoustically different stimuli, the difference waveform is derived from responses to a physically identical stimulus that had the function of a deviant minus when it had the function of a standard (see Jacobsen and Schröger, 2003).

Per participant and per block, we averaged the epochs of the three short stimuli and the epochs of the three long stimuli. Per participant, two difference waves were derived by subtracting responses to physically identical standard from deviant stimuli: namely, the average waveform of short standards (from one block) was subtracted from the average waveform of short deviants (from the other block), and the average waveform of long standards was subtracted from the average waveform of long deviants. There was thus a within-subject factor “duration-type” with two levels, namely short and long, referring to the comparison of short standards with short deviants from reversed blocks, and long standards with long deviants from reversed blocks, respectively. Recall that in the first block of EEG recording, half of the participants per language were presented with short deviants among long standards, while the other half of participants were presented with long deviants among short standards. We therefore also included the between-subjects factor “first-deviant-duration” with two levels: short and long, which refers to the duration-type of deviants from the first block.

We obtained separate grand-average difference waveforms for each experimental condition (i.e., for each combination of first-deviant-duration, vowel-quality, and duration-type). In these grand-average difference waveforms, we searched for a negative peak (“grand peak”) within a large time window between 200 and 360 ms after stimulus onset at the channel Fz; this large window was chosen because MMN effects typically occur 100–250 ms after the onset of deviation, which in our experiment could have been perceived already at about 200 ms after the onset of stimulus (recall that the shortest stimulus was 118 ms). The latency of the grand peak was thus identical for all the 9 subjects in every experimental condition. Subsequently, in a 40-ms window centered at the detected grand-peak, we measured the mean MMN amplitude at every channel for each individual subject. This mean individual amplitude served as our measure of “MMN amplitude” that was submitted to statistical analyses. For statistical tests α was set at 0.05.

## Results

**FIGURE 2:**
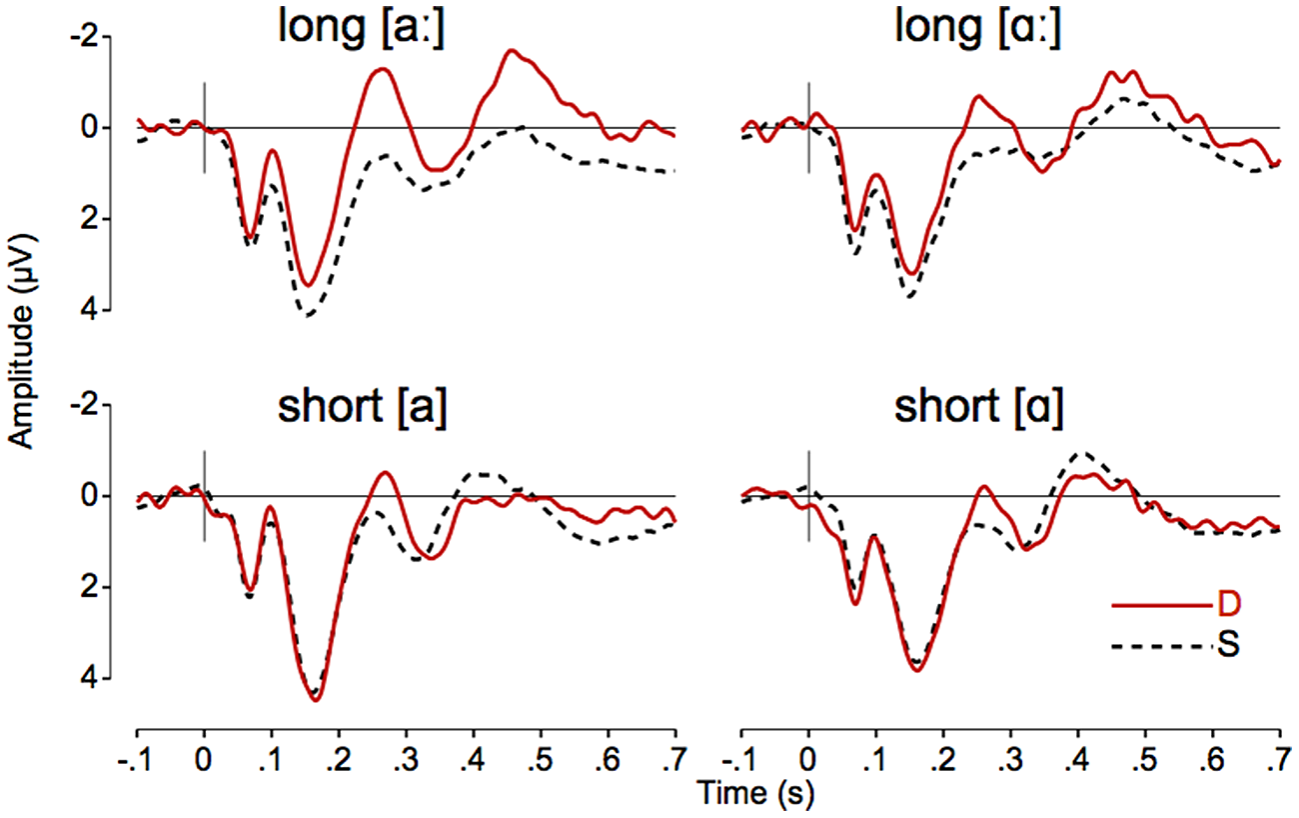
**Grand-average deviant (red solid line) and standard (black dashed line) waveforms at Fz in the two vowel qualities for long **(top)** and short stimuli (bottom)**.

**FIGURE 3:**
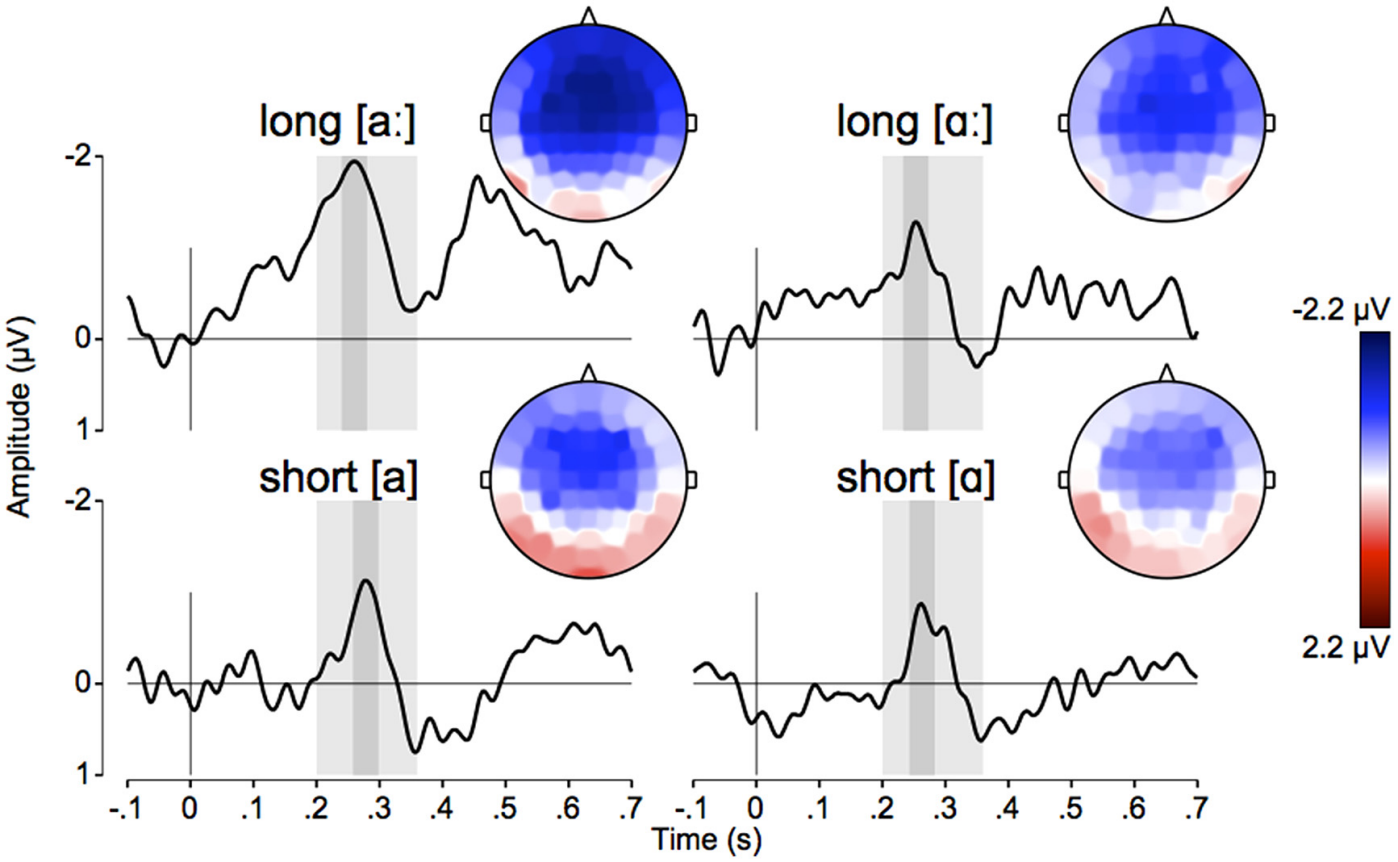
**Grand-average difference waveforms at Fz and scalp distribution of the MMN in the two vowel qualities for long **(top)** and short stimuli (bottom).** The window between 200 and 360 ms in which we searched for grand-peaks are shaded in light gray. The grand peaks for the four respective conditions were detected at the following latencies relative to stimulus onset: 259 ms for long [a**ː**], 278 ms for short [a], 254 ms for long [**ɑː**], and 263 ms for short [ɑ]. The 40-ms time-windows around the grand peaks, in which we measured the average MMN amplitude, are shaded in dark gray. The scalp distributions, accordingly, plot the average MMN amplitudes measured over the 40-ms window.

**Table 1** shows the MMN amplitudes at Fz from block 1 and block 2 for short and long stimuli separately. **Table 2** list the mean MMN amplitudes from block 1 for short and long stimuli, and for each vowel quality, averaged across nine sites: Fz, FCz, Cz, F3, F4, FC3, FC4, C3, C4 (note that the MMN is typically largest over fronto-central scalp sites). **Figure 2** shows the grand average standard and deviant waveforms at Fz, and **Figure 3** shows the difference waveforms at Fz as well as the topographical MMN distributions for each vowel-quality and duration-type. Note that the amplitude of the MMN typically reflects the strength of auditory change detection, i.e., listeners’ perceptual sensitivity to the auditory change: the more negative the MMN amplitude, the stronger the change detection (see e.g., Näätänen, 2001). The MMN amplitude (computed with the approach described in Section “ERP Extraction and MMN Assessment”) is therefore the measure that we assess in our statistical analyses reported below.

**Table 1:** Mismatch negativity amplitude (in μV) at Fz from block 1 and from block 2 for short and long stimuli.

First deviant duration (between-subjects)	Duration type (within-subjects)	Block	MMN amplitude	
			Mean	95% conf. interval
Long	Long	1	-1.486	-2.040…-0.932
	Short	2	-0.069	-0.449…0.311
Short	Long	2	-0.333	-0.887…0.221
	Short	1	-0.841	-1.221…-0.461

**Table 2:** Mismatch negativity amplitude (in μV) from block 1 averaged across nine sites (Fz, FCz, Cz, F3, F4, FC3, FC4, C3, C4).

Duration type	*n*	MMN amplitude: mean and CI			
		[a]		[ɑ]	
Long	9	-1.714	(-2.357…-1.070)	-1.034	(-1.663…-0.406)
Short	9	-0.972	(-1.615…-0.329)	-0.676	(-1.305…-0.047)
Average across long and short	18	-1.343	(-1.798…-0.888)	-0.855	(-1.300…-0.411)

The table shows means and 95% confidence intervals (CIs) per vowel-quality and duration-type, and the number of subjects (*n*) in each group.

First, we ran an exploratory repeated-measures analysis of variance (ANOVA) on the MMN amplitude measured at Fz. This first ANOVA had vowel-quality and duration-type as the within-subject factors, and first-deviant-duration as the between-subjects factor. There was a significant two-way interaction of duration-type and first-deviant-duration [*F*(1,16) = 12.293, *p* = 0.003, *r* = 0.66]. As can be seen in **Table 1**, pairwise comparisons of the means revealed that the average MMN in participants who were first presented with *long* deviants was -1.486 μV for *long* stimuli and -0.069 μV for *short* stimuli (with the 95% confidence interval [CI] of the latter not significantly different from 0). The average MMN in participants who were first presented with *short* deviants was -0.841 μV for *short* stimuli and -0.333 μV for *long* stimuli (with the CI of the latter not significantly different from 0). That is, the MMN was considerably larger for deviants from the first block than for deviants from the second block. This finding replicates the block-effect reported in Chládková et al. (2013), where the attenuation of MMN to deviants presented in the second block was interpreted as a result of habituation to the frequently repeated standards in the first block (McGee et al., 2001). Since the declined MMN responses from block 2 may not reliably represent the listeners’ true sensitivity to duration, we follow Chládková et al. (2013), and further compare the MMNs elicited by deviants from the first block only (**Table 2**; **Figures 2** and **3** accordingly show MMN to deviants from the first block).

A second repeated-measures ANOVA was carried out with vowel-quality ([a] vs. [ɑ]) as the within-subjects factor and with duration-type (short vs. long) as the between-subjects factor. The MMN amplitudes measured at nine channels (Fz, FCz, Cz, F3, F4, FC3, FC4, C3, C4) were included in the analysis, and therefore anteriority (frontal: Fz, F3, F4; fronto-central: FCz, FC3, FC4, central: Cz, C3, C4) and laterality (midline: Fz, FCz, Cz; left: F3, FC3, C3; right: F4, FC4, C4) were also within-subject factors. The analysis revealed a main effect of vowel quality [*F*(1,16) = 4.665, *p* = 0.046, *r* = 0.48]. Pairwise comparisons showed that duration changes in [a] yielded a larger MMN than duration changes in [ɑ] by on average 0.488 μV (95% CI of the difference = 0.009…0.967 μV). The analysis did not detect any other significant main effects or interactions.

## Discussion

This study tested the hypothesis that speakers of a language that appears to use duration to differentiate (some of) its vowels, have abstract representations for ‘short’ and ‘long’ in their phonology. To that end, we examined whether or not Dutch listeners are equally sensitive to duration changes in the vowels /aː/ and /ɑ/ that distinguish Dutch words such as *maan* and *man* (‘moon’ and ‘man’). These two vowels are by some phonological theories described as long and short, respectively (e.g., Moulton, 1962), and in line with that they are usually produced with long and short durations, respectively (Adank et al., 2004). However, when perceiving /aː/ and /ɑ/ Dutch listeners do not always rely on the durational properties of the vowels (e.g., Escudero et al., 2009), and if they do, they seem to notice mispronunciations of duration more readily for words containing /aː/ than they do for words containing /ɑ/ (see van der Feest and Swingley, 2011). Because of the production-perception discrepancy, it is unclear whether /aː/-/ɑ/ is encoded as a length contrast in the mental lexicon of Dutch speakers. The present study tested whether Dutch encodes /ɑ/-/aː/ as a short–long contrast in its phonology.

We reasoned that if the Dutch /ɑ/-/aː/ contrast were phonologically represented as a ‘short–long’ contrast, duration would be an equally important phonetic cue to both members of this contrast, and one would expect to find an equally strong mismatch response to duration changes in both vowels. We measured the amplitude of the mismatch response to duration changes for [a] and [ɑ]. The results showed that duration changes in [a] elicited a larger MMN response than did duration changes in [ɑ], which indicates that Dutch listeners do not rely on duration to the same extent for both members of the /ɑ/-/aː/ vowel pair. Crucially, this result can be compared to MMN responses to duration changes in native vowels that were previously reported for listeners from quantity languages and listeners from non-quantity languages. Specifically, our Dutch listeners’ MMN amplitude for duration changes in [a] was comparable to that of Czech (i.e., quantity) listeners, whose language unequivocally represents vowels in terms of abstract length categories. In contrast, our Dutch listeners’ MMN amplitude for duration changes in [ɑ] was comparable to that of Spanish (i.e., non-quantity) listeners whose language does not have any phonological representations for length (see Chládková et al., 2013). This comparison further indicates that Dutch listeners may use vowel duration differently for /aː/ than for /ɑ/: for the former, they have a strong, *quantity-language-like* reliance on duration, while for the latter they have a weak, *non-quantity-language-like* reliance on duration.

Our finding that Dutch listeners have larger sensitivity to duration changes in a stimulus which has the quality of [a] than in a stimulus which has the quality of [ɑ] indicates that duration is a reliably less relevant phonetic property for the Dutch vowel category /ɑ/ than it is for /aː/. We propose that this differential sensitivity may be represented phonemically: /aː/ is stored as a long vowel, while /ɑ/ does not have any stored specification for vowel duration. The differential phonemic status of length for /aː/ than for /ɑ/ could explain why these vowels are produced with distinct durations. That is, since /aː/ is represented as ‘long’, it is produced with a long duration, while /ɑ/ – which has no length representation – can be produced with any duration, but its short version is most common because it involves less articulatory effort (Boersma, 1998: 149–151; in that respect, it is worth mentioning that, as observed by Lipski et al., 2012: 642, speakers who do not use duration phonemically, e.g., Spanish, tend to realize all vowels with duration values similar to those of Dutch phonetically short vowels: compare for instance the values in Adank et al., 2004 for Dutch and Chládková et al., 2011 for Spanish). The proposed differential phonemic relevance of duration for the two Dutch vowels also explains previous behavioral findings showing that the perceived identity of the stimulus was more likely to be affected by duration changes in [a] than in [ɑ] (e.g., van der Feest and Swingley, 2011). That is, since duration is relevant for /aː/ but not for /ɑ/, listeners perceive a phonemic difference between [a] and [aː] but not between [ɑ] and [ɑː].

Our proposal that only one member of the Dutch /aː/-/ɑ/ contrast is phonologically specified for length is in line with previous studies that found asymmetries in speech perception and explained them by a lack of phonological specification for one member of the speech sound pair in question (e.g., Eulitz and Lahiri, 2004; Mugitani et al., 2009; Roberts et al., 2014). For instance, Roberts et al. (2014) showed that Bengali listeners, whose language distinguishes short and long consonants, accept a lengthened token of a short consonant as a rendition of the short category, but not vice versa. Roberts et al. (2014) explained their result by an asymmetrical phonological specification for Bengali short–long consonant pairs: namely, that a long version of consonant contains all the information necessary for activating a representation of a short category, but that a short version of a consonant misses the durational specification required for a long category. Such asymmetry in phonological encoding is similar to the one proposed here for the Dutch /aː/-/ɑ/ contrast, and it remains a question for future research whether this asymmetry applies across all short–long vowel pairs in Dutch, or in other languages.

In sum, the present study found that Dutch listeners have a reliably larger MMN amplitude to duration changes in [a] than in [ɑ], which disconfirms the hypothesis that the durational difference between Dutch /ɑ/ and /aː/ is encoded in terms of a binary phonological distinction ‘short’ versus ‘long.’ Rather, it is likely that vowel duration in Dutch is a phonemic property specific to certain vowels. This finding demonstrates that speakers of a language such as Dutch, who in their speech production tend to distinguish vowels on the basis of their duration, may not have abstract representations for binary length contrasts – such as ‘short’-‘long’ – encoded in their phonological lexicon.

## Conflict of Interest Statement

The authors declare that the research was conducted in the absence of any commercial or financial relationships that could be construed as a potential conflict of interest.
